# Does Apoptosis Play a Role in Varicella Zoster Virus Latency and Reactivation?

**DOI:** 10.3390/v4091509

**Published:** 2012-09-11

**Authors:** Stephanie F. James, Ravi Mahalingam, Don Gilden

**Affiliations:** 1 Department of Neurology, University of Colorado School of Medicine, Aurora, CO 80045, USA; Email: stephanie.2.james@ucdenver.edu (S.F.J.); ravi.mahalingam@ucdenver.edu (R.M.); 2 Department of Microbiology, University of Colorado School of Medicine, Aurora, CO 80045, USA

**Keywords:** varicella zoster virus, apoptosis

## Abstract

Varicella zoster virus (VZV) is an exclusively human highly neurotropic alphaherpesvirus. To date, VZV has been shown to induce apoptosis, primarily through the intrinsic pathway in different cell types, except for neurons in which the virus becomes latent. This review summarizes current studies of varicella-induced apoptosis in non‑neuronal cells. Future studies are proposed to determine whether apoptosis is terminated prematurely or even begins in neurons that are non-productively infected with VZV.

## 1. Introduction

Programmed cell death, *i.e.*, apoptosis, is tightly regulated and acts to eliminate cells that no longer perform their designated function due to DNA mutations, irreparable damage or virus infection. During apoptosis, caspases (cysteine proteases) are activated that cleave DNA and lead to cell death [1]. Common morphological features seen in apoptotic cells include membrane blebbing and chromatin condensation. Multiple DNA and RNA viruses, including varicella zoster virus (VZV) and simian varicella virus (SVV), induce apoptosis *in vivo* and *in vitro *[2]. VZV is a highly neurotropic, exclusively human alphaherpesvirus. Primary VZV infection, usually in children, produces varicella (chickenpox). Varicella is characterized by a maculopapulovesicular rash that begins centrally on the face or chest and spreads peripherally to the extremities. Rash is often accompanied by fever and fatigue and resolves in 7–10 days. Virus then becomes latent in neurons of cranial nerve ganglia, dorsal root ganglia and autonomic ganglia along the entire neuraxis [3,4,5]. With advancing age or immunosuppression (as in organ transplant recipients or patients with cancer or AIDS), a reduction in VZV-specific cell-mediated immunity results in virus reactivation and zoster (shingles). 

Zoster is characterized by dermatomal distribution pain that lasts for many weeks. Unfortunately, pain often persists for months or years (postherpetic neuralgia) after zoster. Other serious neurological complications of zoster include meningoencephalitis, myelitis, vasculopathy, zoster paresis and retinitis [6,7]. Prevention of VZV reactivation awaits a better understanding of the virus-host relationship in neurons compared to non-neuronal cells. 

Although there is no satisfactory model for VZV pathogenesis or latency in rodents or primates, VZV has a counterpart in the alphaherpesvirus SVV. Like VZV, SVV infection of non-human primates causes varicella, after which virus becomes latent in ganglionic neurons and reactivates to produce zoster [8,9]. VZV and SVV antibodies cross-react, and the viruses share more than 70% nucleic acid sequence homology. 

## 2. Mechanisms of Apoptosis

Unlike necrosis (death due to extracellular trauma), apoptosis of infected cells limits virus spread and thus may be beneficial to the host [10]. Programmed cell death can occur through the extrinsic or intrinsic pathway. In the extrinsic pathwaythe extracellular protein Fas, a member of the tumor necrosis family (TNF), binds to its associated receptor. Upon ligand-receptor binding, the cytosolic portion of the receptor trimerizes and activates the Fas-associated death domain (FADD) which then complexes with the Fas ligand. The resulting Fas/FADD complex cleaves pro-caspase 8, which in turn activates caspase 3. When caspase 3 is cleaved, it enters the nucleus and degrades cellular DNA. Degraded DNA triggers activation of poly (ADP-ribose) polymer (PARP) [11]. PARP then exits the nucleus and targets mitochondria, where it causes release of apoptosis-inducing factor (AIF) into the nucleus, leading to DNA condensation and fragmentation [12].

The intrinsic pathway of apoptosis is triggered by intracellular signals. This pathway is mediated by members of the Bcl-2 superfamily that includes pro- and anti-apoptotic proteins. Anti-apoptotic proteins are Bcl-2, Bck-xL and Bcl-w; pro-apoptotic proteins include Bid, Bax, BAD, Bak and Bok [13]. Pro-apoptotic proteins mediate translocation of cytochrome C from inside the mitochondrial membrane to its outer layer. After release from mitochondria, cytochrome C activates caspase-9, which in turn activates caspase-3, resulting in DNA degradation [14]. Note that cleavage of caspase 8 during the extrinsic pathway may also activate Bid and trigger the intrinsic pathway. Thus, caspases 8, 3 and PARP are useful markers of apoptosis.

Other cell signaling pathways can also affect apoptosis. Because of their potential role in VZV pathogenesis, the extracellular signal-regulated protein kinases 1 and 2 (ERK1/2) in the family of mitogen-activated protein kinases (MAPK), represent a pathway of particular interest. This pathway is initiated by an extracellular growth factor that binds to its receptor. This binding triggers a cell signaling cascade in which cytosolic Ras protein converts GDP into GTP and phosphorylates Raf. Raf then phosphorylates MAP kinase MEK1/2. Finally, MEK1/2 phosphorylates ERK1/2, which then acquires various effector functions, including activation of transcription factors that mediate cell proliferation [15] and enhance viral spread.

## 3. VZV and SVV Can Induce Apoptosis

VZV induces apoptosis in African monkey kidney Vero cells, human melanoma MeWo cells, human fibroblasts and peripheral blood mononuclear cells in culture [16,17,18,19]. Importantly, levels of anti-apoptotic Bcl-2 are reduced (confirmed by both RNA and protein expression) in VZV-infected, but not uninfected cells [16,17,18,19]. Similarly, in SVV-infected Vero cells, levels of cleaved caspase 3 and PARP are increased, while bcl-2 mRNA and protein expression are decreased [20], indicating induction of the intrinsic pathway. However, because cleaved caspase 8 is increased in VZV-infected MeWo cells, the extrinsic pathway is also involved in VZV-induced apoptosis [19]. Because this increase was found in cultures where not every cell was infected, it is possible that VZV- or SVV‑infected cells release proteins such as Fas that act on adjacent uninfected cells. 

## 4. VZV and SVV Can Also Inhibit Apoptosis

Although VZV- and SVV-infected cells become apoptotic, thus limiting spread of virus, studies of VZV proteins revealed that they can increase cellular proliferation and viral replication. For example, the protein encoded by VZV open reading frame (ORF) 12 activates AP1, a transcription factor that increases cellular proliferation [21]. A role for VZV ORF12 in inhibiting apoptosis is suggested by increased levels of cleaved caspase 3 and PARP in cells infected with a recombinant VZV vaccine strain (rRoka) lacking ORF12, and by a greater percentage of rRoka-infected cells expressing the apoptosis-associated cell surface protein annexin compared to parental Roka-infected cells; moreover, because luciferase activity is increased in human embryonic kidney 293 cells expressing AP-1 fused to a luciferase gene after transfection with an ORF12-containing plasmid, ORF12 protein likely promotes proliferation and supports virus survival; addition of an MEK1/2 inhibitor to these cells reduces luciferase activity, suggesting that ORF12 augments MEK1/2 phosphorylation of ERK1/2 to drive AP‑1 [22]. 

Since VZV becomes latent in neurons, the question arises whether apoptosis is terminated prematurely so that VZV does not kill neurons, or whether the apoptotic cascade even begins. Although VZV transcripts and proteins have been detected in latently infected human ganglia, there are no morphological changes in neurons to suggest apoptosis [23]. While no increase in apoptotic marker levels was noted in VZV-infected human fetal ganglionic neurons compared to fibroblasts [17], definitive conclusions cannot be drawn because the “neuronal” cultures were only ~80% pure and likely contained ~20% non-neuronal cells that would have become apoptotic. More recently, differentiated human neural stem cells in cultures containing over 90% neurons (based on staining with neuronal markers) were shown to have significantly less active caspase 3 after infection with VZV than was found in VZV-infected fibroblasts [24]. Further studies in VZV-infected cultures of “pure” neurons are sorely needed. 

VZV ORF63 is the most prevalent and abundant transcript found in latently infected human ganglia [25]. Increased apoptosis was observed in human neurons infected with the parental Oka strain of VZV compared to ORF63 or ORF70 deletion mutants; furthermore, transfection of infected rat neurons with a plasmid containing VZV ORF63 resulted in decreased apoptosis compared to mock‑transfected cells, suggesting that VZV IE63 protein suppressed apoptosis in these cultures [26]. Once again, the “purity” of neurons in those cultures was based solely on morphology, thus no definitive conclusions can be drawn.

Finally, while miRNAs can regulate pro- and anti-apoptotic gene expression levels through transcriptional degradation or translational control, VZV-specific miRNAs have not been detected in human ganglia latently with VZV [27]. 

## 5. Future Directions

The fact that VZV reactivation produces multiple serious neurological disorders underscores the need for a more complete understanding of the VZV-host relationship in neurons and non-neuronal cells. We are currently searching for markers of early and late stages of the apoptotic cascade, as well as for markers indicative of autophagy in VZV-infected neurons in cultures that do not exhibit a cytopathic effect after experimental infection. We are also using VZV ORF63 mutant viruses to determine if VZV IE63 is anti-apoptotic *in vitro*. Finally, our laboratory has generated an SVV ORF63 mutant that can be used to study the role of varicella IE63 *in vivo*.
